# Ginger-Enriched Honey Attenuates Antibiotic Resistant *Pseudomonas aeruginosa* Quorum Sensing Virulence Factors and Biofilm Formation

**DOI:** 10.3390/antibiotics12071123

**Published:** 2023-06-28

**Authors:** Wen-Jie Ng, Chin-Lu Hing, Choon-Boq Loo, Ee-Khang Hoh, Ian-Lung Loke, Kah-Yaw Ee

**Affiliations:** 1 Department of Allied Health Sciences, Faculty of Science, Universiti Tunku Abdul Rahman, Kampar 31900, Malaysia; 2 Centre for Biomedical and Nutrition Research, Universiti Tunku Abdul Rahman, Kampar 31900, Malaysia; 3 Department of Agricultural and Food Science, Faculty of Science, Universiti Tunku Abdul Rahman, Kampar 31900, Malaysia; eeky@utar.edu.my; 4 Centre for Agriculture and Food Research, Universiti Tunku Abdul Rahman, Kampar 31900, Malaysia

**Keywords:** honey, ginger honey, quorum sensing, virulence factors, biofilm formation, *Pseudomonas aeruginosa*, antibiotic resistance

## Abstract

Quorum sensing (QS) in *Pseudomonas aeruginosa* plays an essential role in virulence factors, biofilm formation as well as antibiotic resistance. Approaches that target virulence factors are known to be more sustainable than antibiotics in weakening the infectivity of bacteria. Although honey has been shown to exert antipseudomonal activities, the enhancement of such activity in ginger-enriched honey is still unknown. The main objective of this study was to determine the impacts of honey and ginger-enriched honey on the QS virulence factors and biofilm formation of antibiotic resistant *P. aeruginosa* clinical isolates. Outcomes showed honey and/or ginger-enriched honey significantly reduced the protease activity, pyocyanin production and exotoxin A concentration of the isolates. The swarming and swimming motility together with biofilm formation in all clinical isolates were also significantly inhibited by both honey samples. Notable morphological alteration of bacterial cells was also observed using scanning electron microscopy. A principal component analysis (PCA) managed to distinguish the untreated group and treatment groups into two distinct clusters, although honey and ginger-enriched honey groups were not well differentiated. This study revealed the effectiveness of honey including ginger-enriched honey to attenuate QS virulence factors and biofilm formation of *P. aeruginosa*.

## 1. Introduction

*Pseudomonas aeruginosa* is a highly opportunistic pathogen known to cause severe nosocomial infections in immunocompromised and critically ill patients. The ability of pathogenic bacteria to produce virulence factors, combined with its increasing resistance to multiple antibiotics, has made chronic infections difficult to treat [1]. The World Health Organization (WHO) has identified *P. aeruginosa* as one of the three critical priority pathogens that urgently require new antibiotics [2]. Quorum sensing (QS) is a cell-to-cell communication system that allows bacteria to coordinate their behaviours in response to changes in population density [3]. *P. aeruginosa* uses two QS systems which are the acyl-homoserine-lactone (AHL) *LasR*/*RhlR* network and the 4-hydroxy-2-alkylquinolines (HAQs) *MvfR* network to modulate its virulence factors [4,5]. Thus, *P. aeruginosa* is armed with a variety of virulence factors that can be divided into three main categories, namely bacterial surface structures such as flagella, pilli and lipopolysaccharides that contribute to adhesion or colonization to the host; secretion systems that deliver toxins into the host, such as proteases that degrade proteins in host tissues, pyocyanin that suppress immune response and damage host cells, exotoxin A that inhibit protein synthesis resulting in cell death; and bacterial cell-to-cell interaction including QS and biofilm that confer bacterial communication and drug resistance [1]. As such, strategies that aim to reduce pseudomonal infectivity by neutralizing or weakening bacterial virulence factors have advantages over conventional antibiotic therapy [5]. By interfering with the bacterial virulence, the bacteria will become more vulnerable to the immune system or antibiotics, with minimal selective pressure on the survival of bacteria, thus making it less likely to induce drug resistance [6]. 

In recent years, the use of natural products such as honey and ginger has attracted attention as an alternative approach to managing bacterial infections. Honey has been shown to possess antibacterial properties against various pathogenic bacteria, including antibiotic-resistant strains [7,8,9]. With osmotic pressure, acidity and the presence of hydrogen peroxide, phenolic acids, flavonoids and other antibacterial compounds, honey was believed to cause morphological changes, membrane potential alterations, bacterial metabolism disruption, QS interruption and biofilm inhibition [5,7,8,9]. Ginger, a widely used spice, has also been found to inhibit the growth and biofilm formation of *P. aeruginosa* [10]. Studies showed two major phytochemical compounds of ginger namely zingerone and gingerol, were able to diminish virulence and biofilm formation of *P. aeruginosa* by inhibiting QS activity [6,11]. 

The noticeable increasing demand on healthy food products in recent years drives the innovation and development of new functional foods or alternatives. One of the common practices in food industry is enrichment of food product by adding functional ingredients into a functional food carrier [12]. For instance, adding spices or herbs into honey has become a common method to enhance the health benefit values of honey products [13,14]. Although previous studies have demonstrated the antibacterial properties of honey and ginger individually [6,7,9,10,11], scientific evidence regarding the impacts of the combination of honey and ginger on virulence factors is still very limited.

Therefore, this study aimed to investigate the potential of combining honey bee honey (*Apis cerana*) and ginger (*Zingiber officinale* Roscoe var. Bentong) as a novel approach to manage pseudomonal infections. By exploring the anti-QS virulence factors and antibiofilm properties of this combination, this study seeks to contribute to the development of new strategies for controlling and managing antibiotic resistant *P. aeruginosa* infections.

## 2. Results

### 2.1. Antibiotic Susceptibility

Together with the *Pseudomonas aeruginosa* ATCC 27853 reference strain, the antibiotic susceptibility profile of clinical isolates is shown in Table 1 [15]. All isolates were resistant to ampicillin. Clinical isolate 1 was also resistant to aztreonam, and clinical isolates 2–4 were found to be resistant to ciprofloxacin. Clinical isolates 2 and 4 showed intermediate susceptibility towards aztreonam.

### 2.2. Growth 

The inhibitory effects of tested honey samples on *P. aeruginosa* isolates are tabulated in Table 2. According to the results shown, honey and/or ginger-enriched honey were able to inhibit all isolates with significant smaller diameter of growth zones. Although there was no significant difference between control (untreated bacteria) and honey for clinical isolates 1, 2 and 4 in skim milk agar, ginger-enriched honey was able to show significant smaller diameter of growth zone in these three clinical isolates. On the other hand, both honey and ginger-enriched honey were able to reduce the diameter of growth zones of all clinical isolates in King A agar significantly. However, the differences of growth zones between honey and ginger-enriched honey in skim milk agar and King A agar were not significant.

### 2.3. Virulence Factors

**Figure 1:**
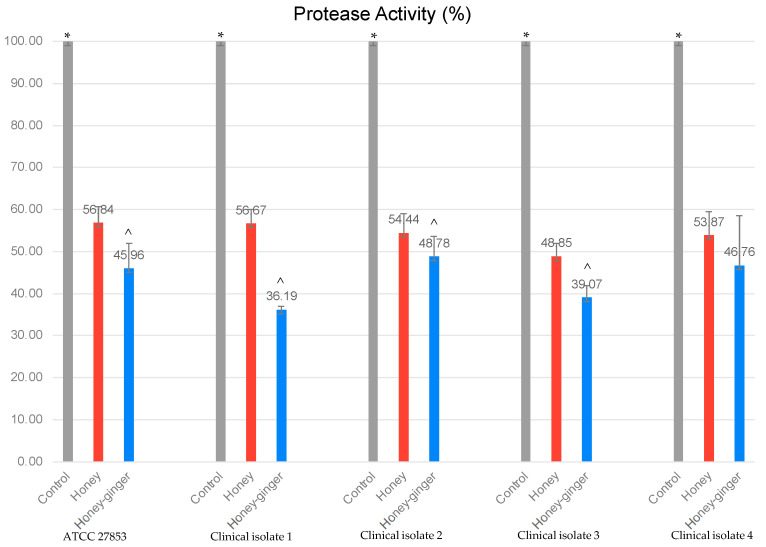
Protease activity of different *P. aeruginosa* isolates was evaluated using azocasein assay. The reduction of protease activity was measured by OD at 400 nm. Control: untreated bacteria; Honey–ginger: ginger-enriched honey. Protease activity is expressed in percentage (%). Data denote mean values of triplicates. * Significance at *p* < 0.05 between control with treatment groups (honey and ginger-enriched honey); ^^^ significance at *p* < 0.05 between ginger-enriched honey and honey.

The impacts of tested honey samples on the QS virulence factors of *P. aeruginosa* were tabulated accordingly. As displayed in Figure 1, the protease activity of all isolates was significantly reduced by both honey and ginger-enriched honey. Both honey samples were also found to reduce the production of pyocyanin in all clinical isolates significantly (Table 3). As shown in the same table, the exotoxin A concentration of two out of four clinical isolates (clinical isolates 1 and 2) was significantly reduced by both honey samples. Significant differences in protease activity and pyocyanin production between honey and ginger-enriched honey were observed in three clinical isolates. However, no significant difference of exotoxin A concentration between honey and ginger-enriched honey was observed.

### 2.4. Motility and Biofilm Formation

**Figure 2:**
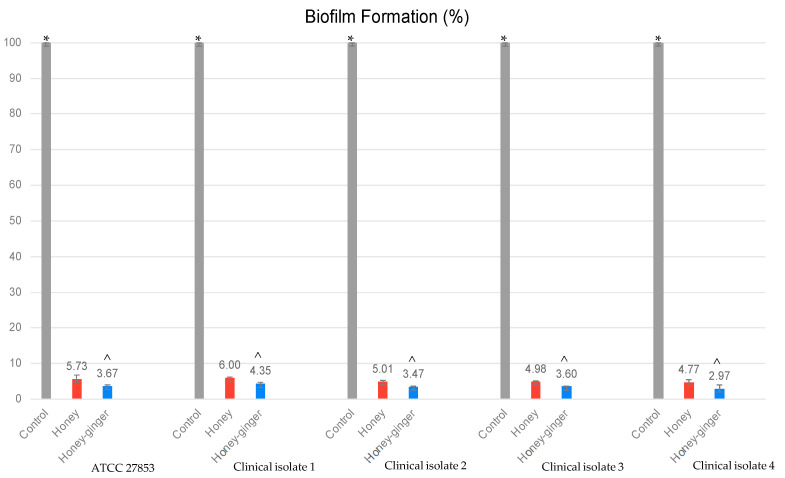
Quantification of biofilm formed in the wells of microtiter plates for different *P. aeruginosa* isolates. The biofilm was quantified at 24 h of incubation by OD at 570 nm. Control: untreated bacteria; Honey–ginger: ginger-enriched honey; biofilm formation is expressed in percentage (%). Data denote mean values of triplicates. * Significance at *p* < 0.05 between control with treatment groups (honey and ginger-enriched honey); ^^^ significance at *p* < 0.05 between ginger-enriched honey and honey.

As shown in Table 4, the presence of honey and ginger-enriched honey significantly reduced the zone of diameter of swarming and swimming zones of *P. aeruginosa*. The swarming motility of clinical isolate 2 was significantly reduced by ginger-enriched honey, but the swarming motility of clinical isolates 1 and 4 were found to be significantly higher in ginger-enriched honey than honey. None of the clinical isolates displayed any swimming motility after treated with ginger-enriched honey. Similarly, as displayed in Figure 2, the biofilm formation was significantly inhibited by both honey samples; and the inhibitory of ginger-enriched honey was found to be significantly higher than honey in all clinical isolates.

### 2.5. Chemometric Analysis

**Figure 3:**
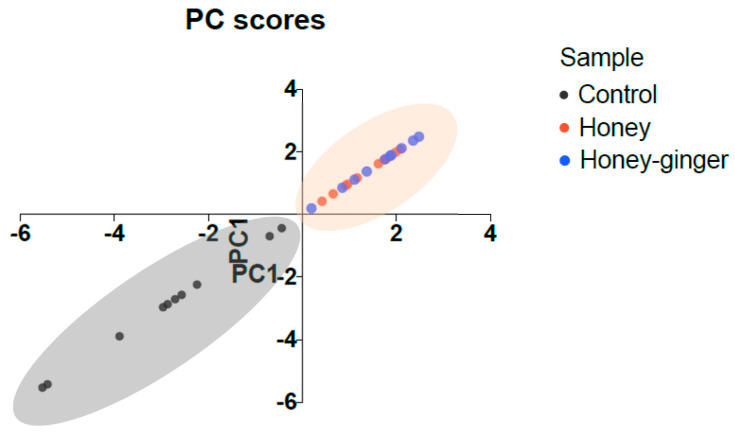
Plot of principal component scores between untreated bacteria (control) and treated bacteria (honey and ginger-enriched honey). The formation of two clusters (grey represents control group; red represents treatment groups) revealed the differences of data obtained from different assays between the control and treatment groups. However, no clear differences were seen between honey and ginger-enriched honey groups. Control: untreated bacteria; Honey–ginger: ginger-enriched honey.

As shown in Table 5, the principal component analysis (PCA) was able to explain a variance of 81.99% with first principal component (PC1) representing 70.09% and PC2 representing 11.90% of the variance. According to the correlation coefficient, the variables that most associated with PC1 were pyocyanin production (−0.958), followed by both swarming and swimming motility (−0.929), protease activity (−0.894), biofilm formation (−0.880), King A agar well diffusion (−0.704), skim milk agar well diffusion (−0.655) and, lastly, exotoxin A concentration (−0.628). Among these variables, pyocyanin production, swarming and swimming motility, protease activity and biofilm formation were the variables associated the most with PC1 (>0.800). Based on Figure 3, this analysis was able to differentiate the control group from treatment groups into two clusters, although honey and ginger-enriched honey groups were not distinguished. The distinct two clusters indicated that the virulence factors and biofilm formation of *P. aeruginosa* were significantly affected by honey and ginger-enriched honey. However, such effects were found not significantly different between honey and ginger-enriched honey, indicating that the impacts of honey on virulence factors and biofilm formation were not significantly enhanced by ginger. 

### 2.6. Morphology

**Figure 4:**
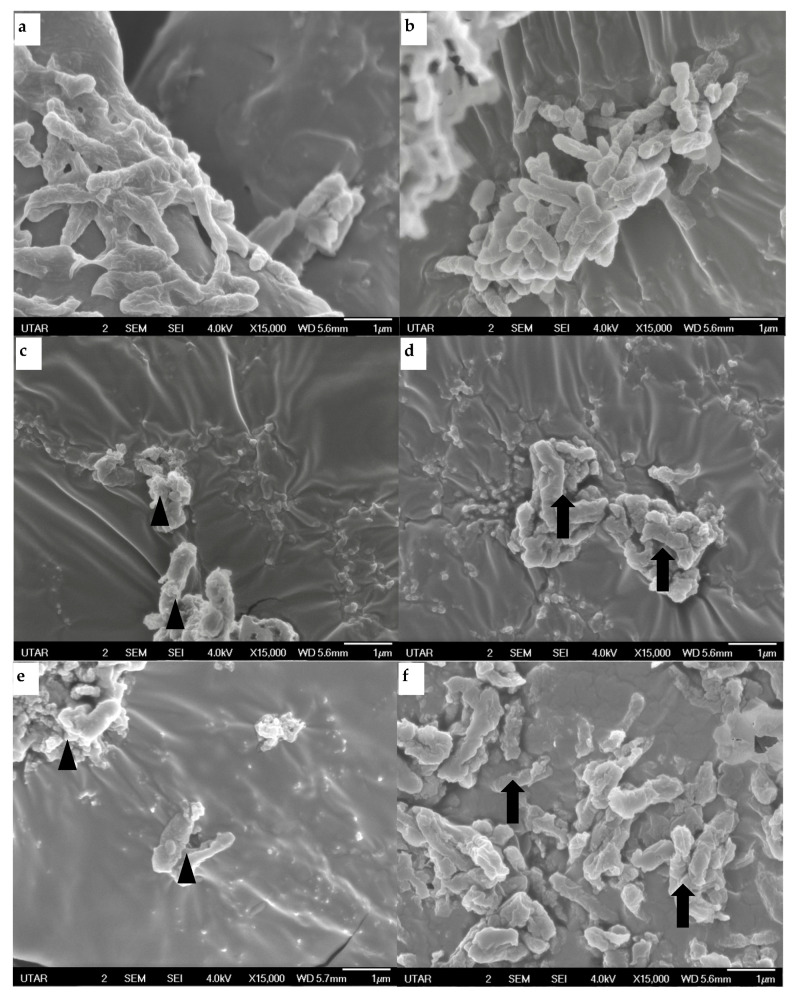
SEM images of *P. aeruginosa* isolates: (**a**) bacteria (clinical isolate 1) without treatment (control); (**b**) bacteria (clinical isolate 3) without treatment (control); (**c**) bacteria (clinical isolate 2) treated with honey; (**d**) bacteria (clinical isolate 4) treated with honey; (**e**) bacteria (clinical isolate 2) treated with ginger-enriched honey; (**f**) bacteria (clinical isolate 3) treated with ginger-enriched honey. The arrowheads and arrows in the images show the significant distortion and rough surface of the bacteria, respectively.

The effects of both honey and ginger-enriched honey on the structure of *P. aeruginosa* were observed using a scanning electron microscope (SEM). As displayed in Figure 4a,b, untreated *P. aeruginosa* were observed to have regular rod shape with smooth surface layers. Furthermore, the untreated cells were connected by extracellular matrix. Cells treated with honey (Figure 4c,d) exhibited remarkable irregular and rough cell surfaces. Similarly, in ginger-enriched honey treated samples (Figure 4e,f), distorted bacteria cells with blebs were observed. In addition, clumping and cell aggregation were observed among treated cells as well.

## 3. Discussion

*Pseudomonas aeruginosa* is recognised as one of the multidrug resistant pathogenic bacteria causing substantial morbidity and mortality worldwide [16]. Together with ATCC 27853 reference strain, the antibiotic susceptibility of clinical isolates was determined (Table 1). Other than ampicillin, which is one of the antibiotics that *P. aeruginosa* resists intrinsically, each clinical isolate was found to be resistant to either aztreonam or ciprofloxacin. *P. aeruginosa* has been shown to possess intrinsic resistance to most antibiotics including β-lactam through restricted outer membrane permeability, efflux system and production of antibiotic-inactivating enzymes [17]. In a study, the highest resistance among 314 *P. aeruginosa* clinical isolates was observed against ampicillin (≥ 98.4%) [18]. Aztreonam is a monobactam antibiotic while ciprofloxacin is a fluoroquinolone antibiotic, and they are used mainly to treat Gram-negative infection, including *P. aeruginosa*. However, based on a study which has collected 924, 740 *P. aeruginosa* isolates from 1997 to 2007, both ciprofloxacin and aztreonam had the highest resistance rate with 28.4% and 20.4%, respectively [19]. Furthermore, 24–27% and 4–7% of *P. aeruginosa* that were isolated from hospitalized patients were found to be resistant to aztreonam or ciprofloxacin, respectively [20,21].

Virulence of *P. aeruginosa* is highly associated with quorum sensing (QS) that regulates virulence factors, motility and biofilm formation [4]. Other than bacteriostatic or bactericidal effects, the ability to reduce virulence of *P. aeruginosa* could be one of the new strategies to combat antibiotic resistant infections [22]. Previous studies have shown the anti-virulence properties of honey and ginger against *P. aeruginosa*. However, such inhibitory effect of the combination of honey and ginger is still unclear [5,6,16]. Other than honey, this study also investigated the ability of ginger-enriched honey to interfere with the QS virulence factors of antibiotic resistant *P. aeruginosa*. 

In this study, skim milk and King A agars were used to screen for the inhibitory effects of honey samples. The formation of halo surrounding growth zones on skim milk agar is due to the breakdown of casein by exoprotease that is released by bacteria [23]. The activity of protease in untreated bacterial isolates was reduced significantly after treated with honey and/or ginger-enriched honey (Table 2). Although no significant difference with honey was observed, complete growth inhibition was seen in clinical isolate 3 after treated with ginger-enriched honey, indicating that the protease activity or the growth of this clinical isolate was suppressed by ginger-enriched honey. On the other hand, King A agar is commonly used to promote the production of pyocyanin but inhibit the formation of fluorescein by bacteria [24]. Thus, the appearance of the blue colour growth zone on the agar is because of the production of pyocyanin, a blue pigment by *P. aeruginosa*. The pyocyanin production of untreated bacterial isolates was reduced significantly after treated with honey and ginger-enriched honey (Table 2). The absence of growth zone observed in clinical 2 could be due to the inhibition on pyocyanin production or the growth of this clinical isolate by honey and ginger-enriched honey. Based on the diameter of growth zones in both assays, the growth of all *P. aeruginosa* isolates was significantly inhibited by the honey and/or ginger-enriched honey. However, ginger-enrichment was not found to enhance the inhibitory effect of honey significantly. To date, this is the first article which used King A agar to screen for the inhibitory impacts of honey on the growth of *P. aeruginosa*.

To further investigate the antipseudomonal effects of honey and ginger-enriched honey, the inhibitory effects on QS regulated virulence factors were determined. For *P. aeruginosa*, acyl homoserine lactone (AHL)-mediated QS involves *las* and *rhl* systems that regulate the production of virulence factors such as protease, pyocyanin and exotoxin and contribute to the formation of biofilm [25]. Protease enzymes participate significantly in the *P. aeruginosa* pathogenesis, degrade host tissues and enhance the bacterial growth and invasiveness [26]. Based on the azocasein assay, the inhibitory ability of both honey and ginger-enriched honey has reduced the bacterial extracellular protease activity of isolates significantly (Figure 1). Furthermore, pyocyanin is known to cause oxidative stress, interfering with the neutrophil-mediated host defence and mitochondrial electron transport [26]. Significant inhibition of pyocyanin production was observed in all isolates treated with honey and ginger-enriched honey (Table 3). The reduction of protease and pyocyanin by honey were also observed in a previous study [5]. Honey prohibited the secretion of extracellular protease that is controlled by las operon in *MvfR* and *lasR* QS systems [27]. The reduction of pyocyanin production was consistent with the inhibition of the *MvfR* QS system. Another study also mentioned zingerone, an active metabolite of ginger that was able to reduce extracellular protease and pyocyanin production of *P. aeruginosa* through the inhibition on *las* and *rhl* systems. It indicated that zingerone may block the downstream signalling pathway by binding with the QS AHL receptor which inhibits the binding of signal molecule with the receptor present in isolates [6]. 

*Pseudomonas* exotoxin A (PEA) is known to be the most toxic virulence factor of *P. aeruginosa* that can inhibit protein synthesis and induce apoptosis in host cells [26]. Together with the reference isolate, the levels of PEA in two out of four clinical isolates were significantly reduced by the honey samples (Table 3). The inhibition of exotoxin A production could be linked to the ability of honey to downregulate the expression of ETA gene together with other QS genes including *lasI*, *lasR*, *rhII* and *rhlR* genes [16].

In this study, both honey and ginger-enriched honey were effective in reducing the swarming and swimming motility of *P. aeruginosa* (Table 4). A study revealed that genes associated with flagella formation were downregulated by honey. This de-flagellation led to reduced motility as well as virulence for *P. aeruginosa* [28]. However, ginger-enriched honey was found to significantly increase the swarming motility of certain clinical isolates more than honey. The increased swarming motility of *P. aeruginosa* with ginger extract was also reported by a study which demonstrated an inverse regulation of motility via flagellar reversal. Such reversal was only found under high viscosity conditions during biofilm formation or swarming but not swimming [29]. The extent of swarming motility was said to determine the final structure of biofilm. When swarming motility is promoted, flat mature biofilm is produced; if swarming motility is inhibited, aggregated biofilm is formed [30]. On the other hand, the swimming motility of all clinical isolates was fully inhibited by ginger-enriched honey. The reduction in swimming as well as swarming motility could be associated with ineffective migration of *P. aeruginosa* that delays biofilm formation [6]. 

Bacterial biofilm is composed of communities of bacteria held together with a self-produced matrix of extracellular polymeric substance (EPS) comprising polysaccharide, protein and environmental DNA [31]. In this study, the significant reduction in biofilm formation by honey and ginger-enriched honey was observed in all clinical isolates (Figure 2). Biofilm reduction was interrelated with swarming and swimming inhibition by honey since motility is essential for the development of biofilm by *P. aeruginosa* [16]. Flagella-mediated swarming and swimming movements are also QS-dependent virulence functions that allow bacteria to attach to surfaces to form biofilm [32,33]. The reduction in the motility was suggested due to the ability of honey to inhibit flagellar movement or even flagellar synthesis [16]. Zingerone was also found to impair the binding of *P. aeruginosa* to surfaces that reduce the biofilm forming capacity significantly [6]. 

A principal component analysis (PCA) was performed to analyse the contribution of variables used in this study. As shown in Table 5, this analysis successfully highlighted the most suitable variables to be used to assess the antibacterial effects of honey and ginger-enriched honey on *P. aeruginosa*. Based on the correlation coefficient, it was suggested that variables including pyocyanin production, swarming and swimming motility, protease activity and biofilm formation were more significant to determining the antibacterial effects of honey and ginger-enriched honey against *P. aeruginosa*. Moreover, this statistical analysis was able to validate the significant changes on the virulence factors and biofilm formation of *P. aeruginosa* due to the effects of honey and ginger-enriched honey (Figure 3).

The antibacterial effects of honey and ginger-enriched honey were further verified by scanning electron microscopy. As displayed in Figure 4, *P. aeruginosa* encountered loss of structural integrity due to the impacts of honey samples. Bacteria cells treated with honey samples were observed to have bloated and filamentous shapes, indicative of the inhibition of septation and cell division [9]. Longer treatment time was identified as one of the key factors to induce membrane injury and lysis in bacteria [34]. Although no significant cellular lysis was observed, widespread structural alteration and damage could make bacteria more vulnerable to antibiotics. It was clearly shown that structural changes in *P. aeruginosa* constituted the mechanism underlying the antibacterial effects of honey.

According to a bibliometric study, the antimicrobial capacity of honey has been correlated with the chemical composition. Phenolic compounds, flavonoids, and hydrogen peroxide were known to influence the antimicrobial activities in different types of honey [35]. At least two mechanisms of action were proposed to relate with the antibacterial effects of honey. The first mechanism is related to the direct biocidal factors of honey that lead to the destruction of bacteria. The other mechanism is about the anti-virulence effects of honey through the inhibitory actions on genes expression, QS, virulence factors production and biofilm formation [5]. This study emphasized the anti-virulence activities of honey samples on *P. aeruginosa* antibiotic-resistant isolates. Results revealed the potential of both honey and ginger-enriched honey to reduce or inhibit the expression of all tested virulence factors of *P. aeruginosa* clinical isolates. It was proposed that the honey samples were able to disrupt bacterial organization and restrict virulence mechanisms [5]. Furthermore, protease activity, pyocyanin production, swarming motility and biofilm formation of *P. aeruginosa* isolates were significantly affected due to the enrichment of ginger in honey. Hence, results suggested that the presence of ginger in honey enhanced the anti-virulence of honey by interfering the QS mechanism, as zingerone has been known to block QS pathways by targeting ligand-receptor interaction [6]. Further studies can be conducted concerning the effects of honey and ginger-enriched honey on the QS virulence factors through blocking QS pathways or reducing the expression of QS genes in *P. aeruginosa*. Furthermore, with the identification of major phytochemicals present in honey and ginger-enriched honey, in silico analysis could be conducted to investigate any possible interaction between active compounds and QS receptors.

## 4. Materials and Methods

### 4.1. Honey Samples

A multifloral honey originated from honey bee *Apis cerana* was harvested from the southern region of peninsular Malaysia. The raw honey samples were added with 18% (*w*/*w*) of ginger (*Zingiber officinale* var. Bentong) dry extract powder. Both honey and ginger-enriched honey samples were kept in glass bottles and stored in the dark at room temperature until use. 

### 4.2. Bacterial Samples

A total of four isolates of *Pseudomonas aeruginosa* were isolated from clinical samples and identified in a hospital located at Pulau Penang, Malaysia. The antibiotic susceptibility of all clinical isolates together with a reference strain ATCC 27853 was screened with ampicillin, gentamicin, ciprofloxacin, imipenem and aztreonam using disc diffusion method. The antibiotic susceptibility profile of each isolate was interpreted using guidelines stated by Clinical and Laboratory Standards Institute [15]. Prior to each assay, bacterial suspension was prepared and adjusted to the concentration of 0.5 McFarland standard (1 × 10^8^ CFU/mL) [36]. 

### 4.3. Agar Well-Diffusion Assay

The inhibition of honey and ginger-enriched honey on the growth of *P. aeruginosa* was assessed using a modified agar well-diffusion assay [9]. Two different agars, namely skim milk agar and King A agar, were used to monitor the inhibitory effect of honey samples on the production of protease and pyocyanin, respectively. For this semi-quantitative analysis, a well with diameter of 6 mm was prepared on the centre of skim milk agar and King A agar. Approximately 50 μL of untreated (control) and treated bacterial suspension was inoculated into the respective well on the agar plates. After incubation at 37 °C for 18 to 20 h, the diameter of clear zone or green zone formed on the respective agar was measured in millimetre (mm). 

### 4.4. Azocasein Assay

The protease activity exerted by *P. aeruginosa* was further quantified in the azocasein assay [37]. Approximately 20 µL of untreated (control) and treated bacterial suspension was added with 250 µL of 1% azocasein and 230 µL of 20 mM TrisHCL. The mixture was incubated at 37 °C for 15 min. Next, 0.5 mL of 10% trichloroacetic acid was added to stop the reaction. After centrifugation at 5000 rpm, the absorbance of the supernatant was read at 400 nm (BMG Labtech FLUOstar^®^ Omega, Ortenberg, Germany). 

### 4.5. Pyocyanin Assay

The amount of pyocyanin produced by *P. aeruginosa* was measured in this assay [3]. Prior to the analysis, pyocyanin production broth (PPB) was prepared, consisting of 2% peptone, 1% potassium sulphate and 0.3% magnesium chloride. The untreated (control) and treated bacterial suspension was added with the PPB in the ratio of 1:10, respectively, followed by incubation at 37 °C for 24 h. In the following day, pyocyanin was extracted by 3 mL of chloroform. The blue colour bottom layer was transferred to another tube and added with 1 mL of 0.2 M hydrochloric acid, yielding a red colour layer on the top. After centrifugation at 6000 rpm, the absorbance of red colour supernatant was read at 520 nm. The concentration of pyocyanin (μg/mL) was calculated by multiplying the absorbance by 17.072.

### 4.6. Exotoxin A Assay

The release of *Pseudomonas* exotoxin A (PEA) was conducted using a sandwich enzyme-linked immunosorbent assay (ELISA) method (Fine Biotech Co., Ltd., Wuhan, China; Cat. No. EH4007). Briefly, together with the standard solutions, diluted untreated (control) and treated bacterial suspension was added into the capture antibody pre-coated well accordingly and incubated at 37 °C for 90 min. After washing, biotin conjugated detection antibody was added into each well and incubated for 60 min. After another washing, horseradish peroxidase-streptavidin conjugate (SABC) working solution was added and incubated for 30 min. About 90 µL of 3,3′,5,5′-tetramethylbenzidine (TMB) substrate solution was added after washing and incubated for 20 min. Lastly, acidic stop solution was added, and the absorbance was read at 450 nm. The concentration of exotoxin A (pg/mL) was obtained after the standard curve was plotted.

### 4.7. Swarming and Swimming Motility Assay

The motility of *P. aeruginosa* was evaluated based on the swarming and swimming activities. Swarming is multicellular wet surface movement powered by rotating flagella while swimming is individual movement in liquid powered by rotating flagella [16]. Prior to each assay, the bacterial suspension was treated with honey and ginger-enriched honey separately (1:1 ratio), and it was incubated overnight at 37 °C. In the preparation of swarming agar, it was composed of 1% peptone, 0.5% sodium chloride, 0.5% agar powder and 0.5% D-glucose. As for the swimming agar, it was prepared as follows: 0.5% peptone, 0.3% yeast extract and 0.3% agar in the nutrient broth. On the following day, the untreated (control) and treated bacterial suspension were point inoculated in the centre of the respective swarming and swimming agar. The plates were incubated at 37 °C for 10 to 12 h. The motility activity of each isolate was determined by measuring the diameter of swarming and swimming zone in the corresponding plate in millimetres (mm). 

### 4.8. Biofilm Inhibition Assay

The ability of honey and ginger-enriched honey to inhibit formation of biofilm was determined by a microtitre plate assay [38]. Each untreated (control) and treated bacterial suspension was added into a well of a flat-bottomed 96-well microtiter plate accordingly. The plate was incubated for the establishment of biofilm at 37 °C for 24 h. After the suspensions were removed, formed biofilms were fixed by methanol and washed with phosphate buffer solution (PBS). Biofilms were then stained with 0.25% crystal violet and washed with PBS. The plate was air-dried in the dark at room temperature overnight. On the next day, the crystal violet-stained biofilm was solubilized by acetone-absolute ethanol (1:1 ratio). The absorbance value of solubilized dye solution was determined at 570 nm.

### 4.9. Chemometric Analysis 

Each assay was carried out in triplicates and conducted at room temperature (23–26 °C) unless stated otherwise. Two-way analysis of variance (ANOVA) was performed to determine the mean value differences at level of significance of 0.05 between untreated group (control) and treatment groups (honey and ginger-enriched honey). Principal component analysis (PCA) was also employed to interpret interdependence and visualize relatedness between data. The statistical analyses were performed using software GraphPad Prism v9.1.1.

### 4.10. Scanning Electron Microscopy 

Morphological changes in *P. aeruginosa* due to the action of honey and ginger-enriched honey were examined microscopically. Prior to processing, each bacterial suspension was incubated with the respective honey sample at 37 °C for 24 h. After centrifugation at 3500 rpm, the obtained pellet was fixed with 2.5% glutaraldehyde in 0.01 M PBS. The pellet was then washed with 0.01 M PBS and, subsequently, with distilled water. The pellet underwent dehydration with ascending concentration of ethanol solutions, starting with 25% ethanol solution followed by 50%, 75%, 95% ethanol solution and, lastly, with absolute ethanol. The dehydrated sample was subjected to freeze drying [9]. Thereafter, the sample was coated with platinum and examined under a scanning electron microscope (SEM) (JEOL JSM-6701F, Akishima, Japan).

## 5. Conclusions

Direct inhibitory effects of honey and ginger-enriched honey against the virulence factors including protease, pyocyanin, motility and biofilm formation of *Pseudomonas aeruginosa*, together with morphological changes, were reported in this study. The PCA also showed two distinguished clusters that differentiated the control (untreated bacteria) and treatment groups (honey and ginger-enriched honey) based on the data obtained in all the assays conducted. Furthermore, the antipseudomonal effects of honey that was enriched with ginger were significantly higher in protease activity, pyocyanin production and biofilm formation. This study demonstrated that honey including ginger-enriched honey can be used as promising anti-virulence agents for the modulation of *P. aeruginosa* antibiotic resistant infections. Further studies into the effects of honey and ginger-enriched honey on QS cellular and molecular targets are necessary.

## Figures and Tables

**Table 1:** Antibiotic susceptibility profile of *P. aeruginosa* isolates.

*P. aeruginosa*	AMP	GEN	IPM	ATM	CIP
ATCC 27853	0 (R)	32 (S)	28 (S)	32 (S)	43 (S)
Clinical isolate 1	0 (R)	34 (S)	31 (S)	15 (R)	26 (S)
Clinical isolate 2	0 (R)	28 (S)	30 (S)	18 (I)	14 (R)
Clinical isolate 3	0 (R)	20 (S)	25 (S)	28 (S)	0 (R)
Clinical isolate 4	0 (R)	24 (S)	25 (S)	19 (I)	17 (R)

Diameter of zone of inhibition was expressed as diameter in mm. R—Resistant; I—Intermediate; S—Susceptible. AMP—ampicillin; GEN—gentamicin; IPM—imipenem; ATM—aztreonam; CIP—Ciprofloxacin.

**Table 2:** Inhibitory effects of honey samples on the growth of *P. aeruginosa*.

*P. aeruginosa*	Skim Milk Agar			King A Agar		
	Control	Honey	Honey–Ginger	Control	Honey	Honey–Ginger
ATCC 27853	18.7 ± 0.6 *	10.3 ± 0.6	7.0 ± 1.0 ^^^	11.7 ± 0.6 *	9.3 ± 1.5	9.0 ± 0
Clinical isolate 1	21.0 ± 1.0 **	22.7 ± 6.7	17.7 ± 2.1	14.0 ± 1.0 *	9.3 ± 1.5	7.3 ± 0.6
Clinical isolate 2	20.0 ± 0 **	18.7 ± 4.6	14.7 ± 1.5	10.3 ± 0.6 *	0	0
Clinical isolate 3	10.7 ± 0.6 *	7.0 ± 0	0	10.7 ± 0.6 *	8.7 ± 0.6	8.3 ± 1.5
Clinical isolate 4	22.3 ± 4.9 **	16.3 ± 0.6	15.0 ± 1.0	35.3 ± 4.7 *	8.0 ± 2.0	7.7 ± 0

Control: untreated bacteria; Honey–ginger: ginger-enriched honey; diameter of zone of inhibition is expressed as diameter in mm. Data denote mean values of triplicates. * Significance at *p* < 0.05 between control with treatment groups (honey and ginger-enriched honey); ** significance at *p* < 0.05 between control and ginger-enriched honey; ^^^ significance at *p* < 0.05 between ginger-enriched honey and honey.

**Table 3:** Effects of honey samples on the pyocyanin production and exotoxin A of *P. aeruginosa*.

*P. aeruginosa*	^a^ Pyocyanin Production			^b^ Exotoxin A Concentration		
	Control	Honey	Honey–Ginger	Control	Honey	Honey–Ginger
ATCC 27853	1.70 ± 0.87	0.74 ± 0.05	1.05 ± 0.36	107.19 ± 11.54 *	46.52 ± 5.33	61.02 ± 3.27
Clinical isolate 1	1.78 ± 0.03 *	0.73 ± 1.07	1.07 ± 0.01 ^^^	95.39 ± 0.91 *	57.44 ± 5.36	67.98 ± 2.98
Clinical isolate 2	1.70 ± 0.02 *	0.92 ± 0.01	0.76 ± 0.02 ^^^	94.55 ± 9.37 *	39.20 ± 2.66	40.46 ± 4.43
Clinical isolate 3	1.72 ± 0.34 *	0.76 ± 0.03	0.80 ± 0.03	39.20 ± 2.06	43.38 ± 2.66	45.27 ± 2.96
Clinical isolate 4	2.74 ± 0.53 *	1.18 ± 0.03	1.05 ± 0.02 ^^^	117.97 ± 16.48	133.68 ± 38.08	117.95 ± 4.89

Control: untreated bacteria; Honey–ginger: ginger-enriched honey; ^a^ Pyocyanin production is expressed as µg/mL of pyocyanin. ^b^ Exotoxin A concentration is expressed as pg/mL. Data denote mean values of triplicates. * Significance at *p* < 0.05 between control with treatment groups (honey and ginger-enriched honey); ^^^ significance at *p* < 0.05 between ginger-enriched honey and honey.

**Table 4:** Effects of honey samples on the motility phenotypes of *P. aeruginosa*.

*P. aeruginosa*	^c^ Motility					
	Swarming			Swimming		
	Control	Honey	Honey–Ginger	Control	Honey	Honey–Ginger
ATCC 27853	28.3 ± 0.6 *	0	1.7 ± 1.2 ^^^	40.0 ± 10.6 *	0	1.7 ± 0.6
Clinical isolate 1	26.3 ± 0.6 *	3.3 ± 0.6	6.0 ± 1.0 ^^^	34.3 ± 0.6 *	6.0 ± 0	0
Clinical isolate 2	25.3 ± 5.9 *	5.3 ± 1.2	3.3 ± 0.6 ^^^	24.3 ± 6.8 *	4.0 ± 1.0	0
Clinical isolate 3	3.3 ± 1.2 *	0	0	5.7 ± 1.5 *	0	0
Clinical isolate 4	39.0 ± 1.0 *	2.3 ± 1.2	6.3 ± 2.1 ^^^	34.0 ± 0 *	0	0

Control: untreated bacteria; Honey–ginger: ginger-enriched honey; ^c^ Swarming (surface motility) and swimming (aqueous motility) are expressed as diameter in mm. Data denote mean values of triplicates. * Significance at *p* < 0.05 between control with treatment groups (honey and ginger-enriched honey); ^^^ significance at *p* < 0.05 between ginger-enriched honey and honey.

**Table 5:** Factor loadings for anti-QS virulence and biofilm formation parameters of honey samples.

Principal Component (PC) Number	PC1	PC2
Eigenvalue	5.607	0.9521
Proportion of variance	70.09%	11.90%
Loading of variable		
Skim milk agar well diffusion	−0.655	
King A agar well diffusion	−0.704	
Protease activity	−0.894	
Pyocyanin production	−0.958	
Exotoxin A concentration	−0.628	
Swarming motility	−0.929	
Swimming motility	−0.929	
Biofilm formation	−0.880	
